# Patient involvement in medical research: what patients and physicians learn from each other

**DOI:** 10.1186/s13023-018-0969-1

**Published:** 2019-01-24

**Authors:** Kalen Young, Dana Kaminstein, Ana Olivos, Cristina Burroughs, Celeste Castillo-Lee, Joyce Kullman, Carol McAlear, Dianne G. Shaw, Antoine Sreih, George Casey, Vasculitis Patient-Powered Research Network, Pablo J. Abonia, Nita Aines, Paul Brown, April Cardone, Michael Fernandez, James Fingar, Carol Gorman, Ida Hakkarinen, Sharon Hatfield, Sean Hennessy, Paul Jacobsen, Tanaz Kermani, Marianne Vennitti, Peter A. Merkel

**Affiliations:** 1 Vasculitis Foundation, Kansas City, MO USA; 2 Organizational Dynamics, Liberal and Professional Studies, University of Pennsylvania, Philadelphia, PA USA; 3 Data Management and Coordinating Center (DMCC), University of South Florida, Tampa, USA; 4 Division of Rheumatology, University of Pennsylvania, Philadelphia, PA USA; 5 Vasculitis Patient-Powered Research Network, Philadelphia, PA USA; 6 Division of Rheumatology and Department of Biostatistics, Epidemiology, and Informatics, University of Pennsylvania, White Building, 5th Floor 3400 Spruce Street, Philadelphia, PA 19104 USA

**Keywords:** Vasculitis, Patient engagement, PCORnet, PCORI

## Abstract

**Background:**

There is increasing interest in actively involving patients in the process of medical research to help ensure research is relevant and important to both researchers and people affected by the disease under study. This project examined the recently formed Vasculitis Patient-Powered Research Network (VPPRN), a rare disease research network, to better understand what investigators and patients learned from working on research teams together.

**Methods:**

Qualitative interviews were conducted by phone with patients, physician/PhD-investigators, and study managers/staff who participated in the network. The question guiding the interviews and observational analysis was: “What have investigators and patients learned about working together while working on VPPRN teams?” Interview transcripts were analyzed in combination with observations from multiple in-person and telephone meetings.

**Results:**

Transcripts and notes were reviewed and coded from 22 interviews conducted among 13 patient-partners, 5 study managers/staff, and 4 MD or PhD-investigators, and 6 in-person and 42 telephone/web-conference meetings. Patient-partners and investigators characterized their working relationships with one another, what they learned from their collaborations, and provided recommendations for future teams of patient-partners and investigators. Major themes included the great benefits of communicating about activities, being open to listening to each group member, and the importance of setting reasonable expectations.

**Conclusions:**

Direct engagement in research design and development by patient-partners and co-learning between investigators and patient-partners can result in a positive and productive working relationship for all members of a medical research team. This bi-directional engagement directly benefits and impacts research design, participant recruitment to studies, and study subject retention.

## Background

There is increasing interest in actively involving patients in the process of medical research to help ensure research is relevant and important to both investigators and people affected by the disease under study. Increasing attention has recently been paid to examining patient engagement in health care research [1]. The Patient-Centered Outcomes Research Institute (PCORI) defines patient engagement as “The meaningful involvement of patients, caregivers, clinicians, and other healthcare stakeholders throughout the entire research process—from planning the study, to conducting the study, and disseminating study results” (https://www.pcori.org/about-us/our-programs/engagement/value-engagement). Patient engagement may be especially important for the study of rare diseases in which the number and size of research studies are relatively small, and the added benefit of more targeted outcomes and facilitated recruitment that patient research partners can add is particularly advantageous. Research on patient engagement has rarely incorporated qualitative methods that involve participants who are directly involved in the research process [2]*.*

This study examined patient engagement in the development and implementation of the recently-formed Vasculitis Patient-Powered Research Network (VPPRN), a rare disease research network, to better understand what investigators and patients learned from working on research teams together. The process and structural framework for engaging patients in research with the VPPRN included: 1) establishing network governance policies and structure that listens to the voices of all stakeholders including patients, physicians, investigators, patient advocacy groups; 2) empowering patients and other stakeholders to take on leadership roles and participate at different levels, optimizing patient and stakeholder expertise, time, and interest; 3) harnessing the broad influence of multi-stakeholder expertise and addressing training needs of all stakeholders. “Patient-partners” include patients, family members, caregivers, and organizations that are representative of the population of interest. Intersecting all aspects of the patient engagement process in research is the learning between investigators and patients. Research on patient engagement has rarely incorporated qualitative research methods that involve participants in the research process [2]. The current report details the results of a qualitative interview and observational study that examined what investigators, research managers, and patients learned from working together on research teams. An overview of the literature on the topic of stakeholder engagement in medical research was conducted and is included in the background section to give context to this research. This study focused on a central and important aspect of this engagement process: learning between investigators and patients.

There has been rising interest in patient and stakeholder engagement in clinical research over the past 10 years [3]. Much of this research has focused on ways to engage stakeholders, logistical arrangements needed to involve patients, and issues such as making sure patients understand medical and research terminology. With this basic research completed it is time to focus attention on the deeper issues involved when investigators and patients work together on research projects. An appraisal of the literature on engaging patients with rare diseases in research found 35 studies. However, none of the studies included an “empirical assessment of engagement practices and their effectiveness” [4]. Using a case study methodology, researchers identified several key principles necessary for patient engagement in research endeavors, including: ensure balance in participating stakeholders, obtain participant “buy-in” to the process and understanding of their roles, provide neutral and expert facilitators for research discussions, establish connections between participants, and keep participants engaged throughout the research process [5]. Another systemic review of research on patient engagement included 142 studies and concluded that “patient engagement increased study enrollment rates and aided researchers in securing funding, designing study protocols and choosing relevant outcomes” [6]; these authors concluded that the most commonly cited challenges were the need for funding to involve patients and the “overarching worry of a tokenistic engagement” of patients [6]. A study most closely related to the current research examined the language of engagement [7]. “We learned that fruitful collaborative work must attend to the creation of a common language, which we refer to as the *language of engagement*”. “We encourage other researchers to think critically about their cultural competency, to be mindful of the social power dynamics between patient and physician, to reflect on how their understanding may differ from those of their patient partners …”.

A report on patient engagement issued by the [8] emphasized the need for consistent terminology, and the need to engage stakeholders early and to maintain relationships, being flexible about the methods of engagement [8]. Another study that includes clear recommendations for patient engagement emphasized that the engagement needs to be “bi-directional between stakeholders and researchers” and emphasized that “… researchers and stakeholders should be committed to the process at the outset; neutral and expert facilitators should be used to guide research discussions; connections among stakeholders should be encouraged; and an environment of mutual respect should be fostered” [9].

Few studies have examined patient engagement from the patients’ perspective. One study that did undertake this focus found that patients were concerned that the process of discussion and deliberation be fair, that patients’ perspectives be taken seriously, and that tokenism be avoided at all costs [10]. The concern about tokenism appears in other research studies on patient engagement [11].

Much of the literature on patient engagement in medical research is prescriptive, and includes learnings from researchers’ experience with involving patients. The existing quantitative or qualitative work in this area is often based on small samples. Only a few studies have closely examined how to develop productive relationships between investigators [12] and patients when involving patients in the research process. The current study sought to further this examination by looking at the relationships that developed between investigators and patient-partners over an 18-month period.

## Methods

This qualitative study examined the results of 18 months of data collection for a project, funded by the PCORI in which patient-partners, physician investigators, PhD investigators, and managers worked on teams building a network of patients for future research projects, to review plans, ideas, and protocols for research studies, and to generate ideas for future research endeavors.

### The Vasculitis Patient-Powered Research Network

Vasculitis is a set of rare organ- and life-threatening diseases of vascular inflammation linked by similar pathophysiologies. Despite improvement in the overall prognosis of vasculitis since the introduction of regimens based on combination immunosuppressive therapy, the cumulative morbidity and mortality from both disease and treatment for most patients, the social impact, and the costs remain high.

The Vasculitis Patient-Powered Research Network (VPPRN) is a collaboration among patients, patient advocacy organizations, academic clinical investigators, expert clinicians, biomedical informaticians, qualitative and quantitative methodologists, and funding organizations, all dedicated to conducting high-quality clinical research in vasculitis (www.vpprn.org). The VPPRN was a founding member of the Patient-Centered Outcomes Research Network (PCORnet, www.PCORnet.org). PCORnet directly engages patients, physician-investigators, research methodologists, and project managers to work together to build a collaborative national resource using the partnership and health data for better research [1]. Patients are fully engaged in the management of the Network with roles that include strategic planning, developing, reviewing, and approving research studies.

The VPPRN embraces the collaborative, patient-centered philosophy of PCORnet and has, from the inception of the Network through its full implementation, involved patient-partners at every level of organizational governance and research planning. The co-principal investigators of the VPPRN are an academic physician-scientist and a patient-partner, and the VPPRN is an extension of an already highly collaborative relationship among the Vasculitis Clinical Research Consortium, the major vasculitis research network, and the Vasculitis Foundation, the major patient advocacy group for vasculitis. Additional patient-partners with vasculitis were chosen by the patient co-PI and Vasculitis Foundation staff through a competitive selection process. Training in patient participation in research was provided to all patient-partners. Patient-partner training was developed in collaboration with consultants from the Organizational Dynamics Program at the University of Pennsylvania and was provided online over multiple training sessions by the VPPRN Network and Data managers.

The VPPRN maintains an on-line research registry through which patients with all forms of vasculitis provide clinical data about their condition. The type of information collected through the VPPRN portal includes data elements relevant to diagnosis, disease extent, medications, demographics, healthcare team, and patient-reported outcomes. Member patients in the VPPRN have consented specifically to take part in research activities.

### Interviews

All patients, physician-investigators, and managers who had participated in the VPPRN governance since 2014 were invited to participate in the study; a total of 22 interviews were conducted. Because of the differences in status and authority (actual or perceived) between patients and physician-investigators, the study was interested in understanding how patients, physician-investigators, PhD-investigators, and study staff worked together. All participants in this study, including patient-partners, investigators, and study staff provided informed consent to participate in the interviews and observations, and to have their data used for publication. Thirteen of 17 patient-partners involved in the Network governance structure participated in this project in 2015–2016 and were interviewed about what they learned from working on teams with physician-investigators. Four of 6 physician/PhD-investigators involved in the network governance structure participated in this project and were interviewed about what they learned from working on teams with patients. The 3 research managers (Network Manager, Project Manager, and Data Manager) were interviewed about what they learned from working on teams with both patients and investigators.

The interviews were conducted using an internet-based conference system and were recorded to ensure accuracy. The interviews were transcribed for coding. Interviewees were asked 11 questions. The questions ranged from what they have found to be most exciting about working on teams with investigators and patients to specific questions about what the interviewees learned from working with those outside of their identity group. Emerging themes and the researchers’ impressions regarding the interactions between physician-investigators and patients, boundaries, norms, roles, leadership and decision-making were documented.

### In-person meetings and teleconference calls

The rich interview data was supplemented with the observational data. The use of observational and interview data helped to gain a deeper understanding of what physician-investigators and patients are learning from working together. Participant researchers attended a total of 74 meetings, 6 were in-person and the rest were by web-conferencing. Official minutes and participant researchers’ observational notes from the 6 in-person meetings and 42 telephone/web-conference meetings were reviewed and coded. Of the 74 meetings the participant researchers attended, 42 were chosen for review and coding. These 42 meetings were chosen for analysis as 2 or more patient-partners participated. Since the focus of this research is on what investigators and patient-partners learn from working together, only the meetings with 2 or more patient-partners involved were included in the analysis.

### Data analysis

The interview and meeting transcripts were reviewed and coded for emergent themes, insights, and patterns, and analyzed utilizing a hybrid method of coding. There were three researchers coding the meetings independently. The hybrid approach included incorporating a priori codes derived from literature references and open, emergent codes arising in the data that were different from the pre-set codes. The same method of coding was applied to the observational documents until data saturation was reached. For the purposes of this research, data saturation was met after the coding of 42 of the meetings and the 13 interviews. The coding scheme was refined and each category defined by breaking down flourishing codes into sub-codes and collapsing other codes into larger themes. Final codes and categories were transferred into a data table.

## Results

### Patient-partner characterization of their working relationships with investigators

When asked about how patient-partners characterized their working relationships with the investigators, over half were positive [5], while 6 had some critical assessments. For teams to work effectively, for trust and learning to occur, members need to feel positive about their working relationships with others on the team. Over half of the patient-partners characterized their relationship with investigators in a fully positive manner and an almost equal number characterized their relationship as moderately positive. Feedback for improvement was garnered from all patient-partners. A patient-partner suggests, “It is important to be explicit about the expectations; due dates for deliverables need to be firm and the outcome of each deliverable needs to be well described.”

The VPPRN fosters a group culture in which stakeholders are empowered with responsibility over Network activities, and are given the tools to maximally contribute to the groups’ goals. Feedback from participants indicated that such empowerment is appreciated but could be expanded upon. Although, patients, clinicians, investigators and other stakeholders may each play different roles at times in Network management, and Network members have varied expertise, all views need space to be shared and are important to consider in the work of the VPPRN.

### What patient-partners learned from working with investigators

Patients’ responses provided few specifics about what they learned from investigators and managers. The lack of specificity is attributed to the task emphasis in the working teams in the Network. To maximize efficiency and capitalize on limited meeting time, the teams focused on the deliverables under their charter. The participants felt that the considerable milestones and time constraints afforded the teams little time to explore each other’s areas of expertise. The patient-partners valued the commitment and contributions of the investigators and managers. Consequently, they had few criticisms about the other stakeholders on the teams; they were more specific in discussing the barriers they experienced in learning from investigators and managers.

### Patient-partners’ recommendations for future teams of patient-partners and investigators

The recommendations that patient-partners made for future teams undertaking this type of work centered mainly on improvements to communication methodologies. Four of the patient-partners expressed difficulty with meetings by conference call, and not being able to see their fellow team members. They indicated that meetings using video and in-person meetings would help to create rapport and build more trust. Three patient-partners mentioned that more effort should be put into involving patient-partners. Two patient-partners talked about the need to set clearer expectations at the start about what would be involved in the team’s work. More information about how the research process works was recommended by one patient-partner and another person urged future teams to have a fuller understanding of the whole (i.e., what other teams in the Network are doing and working on). One person recommended that the discussions be more open, another suggested specific assignments between meetings, and another person emphasized the need for patient-partners to educate the investigators.

### Investigators and managers characterization of their working relationships with patient partners

Investigators and managers were generally positive in characterizing their relationships with patient-partners on the teams they worked on. All nine investigators and managers valued the opportunity to work with patient-partners. The investigators and managers noted feelings of respect and appreciation for the insight and knowledge patient-partners bring to inform the development of VPPRN research protocols.

### What investigators and managers learned from working on teams with patient-partners

Investigators and managers acknowledged the need for improvement when asked to characterize the working relationships between them and the patient-partners. Six investigators and managers expressed interest in working to improve the learning environment that was created on the teams. Investigators and managers also learned ways in which patients’ felt heard and empowered which can only be learned through interaction with each teams’ patient-partners. Investigators and managers also learned the substantial importance of setting expectations and clearly defining roles, deliverables, and timelines.

### Investigators’ and managers’ recommendations for future teams of investigators and patient-partners

The interviewees contributed a range of suggestions for future teams that involve investigators and patients. Four interviewees emphasized the need for recruiting patients who have high levels of confidence and professional demeanor. Two interviewees emphasized the importance of setting clear expectations at the start. One interviewee suggested that investigators be trained on how to engage patients in this type of collaborative endeavor. One discussed the importance of operating using clear team guidelines and principles. Another thought it was important to make sure that everyone understood the jargon and acronyms being used. One interviewee discussed the importance of ensuring that investigators and patients had equal relationships on the teams.

### Working relationships during team meetings

Review of notes and coding were conducted for the 42 team meetings in which 2 or more patient-partners participated. All meetings were led by an investigator or manager. The predominant format was for the investigator or manager to start the meeting, go over the agenda, and then to call on team members, including the patient- partners, and ask for their input and reactions. The resulting data indicated that investigators and patient-partners were clearly respectful of each other; a tone of respect and consideration was present in all 42 meetings. As further evidence of the constructive nature of the team interactions when disagreement did occur it was handled as a learning opportunity. There were no instances of angry or conflictual interactions during the 42 meetings.

### Incorporation of patient-partners’ ideas

There was evidence that patient-partners’ ideas were incorporated into the work of the Network. Each team meeting included at least one example of a patient-partner contributing an idea or reacting to an issue, and the investigator or manager leading the meeting reinforcing that comment positively. For example, an investigator or manager often said, “It is very helpful that you raised that point.”

### Recommendations from patient-partners for improved team dynamics

In four team meetings patient-partners indicated that they wanted more information about what the other teams in the Network were doing. In six team meetings patient-partners indicated that they needed more time to consider the issues being discussed, or that they felt rushed in addressing the issues raised in the meeting. Patient-partners indicated numerous times that they would like to have in-person meetings to get to know each other more fully.

## Discussion

There is sufficient evidence to support the understanding that establishing productive and positive partnerships with multiple stakeholders in research development requires supportive organizational policies and structure that takes into account different power structures and hierarchy*.* The purpose of this qualitative study was to better understand what medical investigators and patient-partners learn from each other by working on teams together. It was based on the premise that for real engagement and involvement to occur in groups where members have different levels of power and status learning needs to be reciprocal [13–15]. Due to the low prevalence and diversity of rare diseases and resulting small number of patients, patient communities for rare diseases tend to be highly motivated to participate in research [16]. This motivation and willingness to participate in research has made rare disease research a fertile space to expand and build upon the benefits of involving patients in the development of research protocols. Patient’s partnering in research development traverse more traditional sociomedical landscapes, intersecting, and in many cases redefining, knowledge and power hierarchies in a research development space.

The teams examined had the goal of creating a network of patients to participate in future research studies, gaining input from patients about the design of research studies, and vetting ideas for future research. Researchers looking at patient engagement in medical research endeavors are now starting to refer to this aspect of the work as "co-learning"[1].

The initial goals of the VPPRN were to involve patients in enrolling patients in the Network who would provide data for future research studies, to develop a secure and user-friendly patient-portal for patient involvement in the VPPRN, to review research forms and surveys, and to vet ideas for future research studies. The goals of the VPPRN are aligned with those of Patient-Centered Outcomes Research Institute to engage patients to conduct rigorous research. The VPPRN has been highly successful in meeting all these goals. There is strong evidence from the interviews with patient-partners, investigators, managers, and observations of the team meetings, that the interaction during the team meetings between investigators, managers, and patient-partners was quite positive. When disagreements occurred, they were used as learning opportunities and were considered constructive to the group process. The meetings were often described as being “respectful” and “cordial”.

In terms of learning, there is ample evidence that patient-partners learned about medical terminology and research issues. Investigator and manager learning was evident in the work products of the Network and reflexivity of team meetings and procedures. Investigators and managers emphasized the need to set expectations and clearly define roles, a theme that emerged from working with patient-partners and learning about their needs in order to meaningfully engage patient-partners. The format of the team meetings, which were led by a manager or investigator, indicates an area for improvement in collaboration. Based on the research on collaboration, shared leadership between patient-partners, investigators, and managers is expected. The structure of the initial meeting leadership may have been more indicative of the newness of the Network and respect for patient-partners’ time commitment. The opportunity to improve balance between collaboration, time commitments, and roles surfaced periodically.

Another important learning from this work is that both patients and investigators are eager to work together and power differences and hierarchies can be bridged. Patients on the teams studied consistently indicated that having a voice in research studies and future research made them feel that they were making a positive contribution. For patients who sometimes feel isolated or inconsequential this boosted their confidence and helped them to see that their knowledge about their disease could benefit others. Investigators consistently appreciated hearing the patient’s perspective and this contributed to their understanding of how to best protect patient’s confidentiality, to increase their likelihood of understanding and completing surveys, and how patient’s view future research priorities.

## Conclusion

While there is ample evidence that the VPPRN teams met their task goals, there are opportunities to improve shared learning between investigators and patients. Co-learning is essential for future efforts to increase patient engagement. A good deal of evidence exits about how to recruit and organize patient involvement in research endeavors. A next important step could be to develop methods to more deeply engage patients and investigators in learning from each other. This study is an important step in that direction, and there is a need for additional studies which probe more deeply into the factors that encourage engagement, trust, and learning between patients and investigators. Such work will hopefully lead to a healthy and positive transformation in how health care research is conceived, designed, and conducted.

## Abbreviations

PCORI: Patient-centered outcomes research institute
PHI: Personal health information
PI: Principal Investigator
VF: Vasculitis foundation
VPPRN: Vasculitis patient-powered research network
